# Causes and consequences of delays in treatment-withdrawal from PVS patients: a case study of *Cumbria NHS Clinical Commissioning Group v Miss S and Ors* [2016] EWCOP 32

**DOI:** 10.1136/medethics-2016-103853

**Published:** 2016-09-23

**Authors:** Jenny Kitzinger, Celia Kitzinger

**Affiliations:** 1 School of Journalism, Media and Cultural Studies, Cardiff University, Cardiff, UK; 2 Department of Sociology, University of York, York, UK

**Keywords:** Decision-making, Human Dignity, Law, Right to Refuse Treatment

## Abstract

Life-extending treatment, in the form of artificial nutrition and hydration, is often provided to people in permanent vegetative states (PVS) in England and Wales for many years, even when their family believes the patient would not want it and despite the fact that no court in the UK has ever found in favour of continuing such treatment for a patient with a confirmed PVS diagnosis. The first half of this article presents a close analysis of the recent case of *Cumbria NHS Clinical Commissioning Group v Miss S and Ors* [2016] EWCOP 32. It examines the causes of delay in bringing this case to court and reaching a final judgment. It draws not only on the published judgment, but also on the two authors' involvement in supporting the family (before, during and subsequent to the court hearings) as a result of their academic and policy-related work in this area. This includes conversations with the family and with members of the clinical and legal teams, and observations in court. The second part of the article draws out the ethical and practical implications of the findings for theory and policy and suggests ways forward in relation to (a) the provision and inspection of care for these patients; (b) legal practice in relation to ‘best interests’ and (c) the perceived requirement under English law for a court application before life-prolonging treatment can be withdrawn from PVS patients—even in the absence of any ‘in principle’ opposition.

## Introduction

After suffering catastrophic brain injuries in August 2012 (at the age of 38), Miss S was given life-extending treatments for nearly 4 years until May 2016. It was then that, following an application from the Clinical Commissioning Group (CCG; with the support of S's family), Hayden J—who heard the case in the Court of Protection—approved the declaration that it was lawful and in her best interests for artificial nutrition and hydration (ANH) to be withdrawn. This case is typical of many other cases concerning patients in a Permanent Vegetative State (PVS) that come before the Court of Protection in that the patient received life-extending treatment, in the form of a feeding tube, for many years. It is *a-typical*, and a ‘landmark’ case, in that, at both the directions hearing and at the substantive hearing, the judges expressed concerns about avoiding unnecessary delays.^i^ In the final judgment Hayden J said:

[T]he avoidance of delay in medical treatment cases is an important imperative […] This is not to say that assessments ought to be rushed or that delays may not sometimes be clinically purposive, but respect for a patient's autonomy, dignity and integrity requires all involved in these difficult cases to keep in focus that these important rights are compromised in consequence of avoidable delay. (para.13)

The nature of the ‘avoidable delay’ was not addressed in court. Hayden J commented that he “found it difficult to understand entirely why this process has taken quite as long as it has” (para 13) but noted that he had not been required to investigate the reasons. That is the task we take on here. Identifying, and examining the causes of, ‘avoidable’ (as opposed to ‘clinically purposive’) delay is important not only because of the ethical consequences for S and for her family but also because this analysis may help to avoid delays for future patients.

## Our involvement

Our involvement in this case came about via our academic work studying the social, legal and ethical aspects of prolonged disorders of consciousness (ie, vegetative and minimally conscious states). We also have personal family experience of catastrophic brain injury and have written—and spoken—publicly about this^1^ and our status as ‘insider’ researchers means that other families have said they feel that we will understand and empathise. In 2014 we used our research to create an online resource^2^ to support families and to provide information for practitioners in this field. Partly as a consequence of this, we have since been contacted by families concerned by what they view as intolerable delays in the process of applying to court for declarations that it would be lawful and in the patients' best interests for artificial nutrition and hydration to be withdrawn.

We have provided informal support for several families in an effort both to assist them and to ensure appropriate medico-legal action for their relatives in compliance with the relevant national clinical guidelines.^3^ Our support has included: pointing family members to key sections of the national guidelines, the Mental Capacity Act 2005 or recent case law; talking directly with members of the patients' clinical or legal teams; visiting the patient; arranging contacts with neuro-rehabilitation and legal experts; and providing direct support (eg, accompanying family members to best interests meetings and to court hearings).

In previous research we explored the experience of a wide range of families with relatives in prolonged disorders of consciousness—some of whom were fighting for treatments to be given, rather than seeking declarations that it would be lawful to withhold them.^2^ ^4^ We subsequently focused specifically on the experience of those families involved in court applications for withdrawal, again drawing on (mostly retrospective) interview accounts.^5^ This article uses a different approach. We examine just one case as it unfolded in real time—thereby enabling a prospective, dynamic and contextual understanding of how delays emerge and their effects on the family at points when subsequent events (and the eventual outcome) have not yet occurred. This longitudinal approach enables “a nuanced understanding of phenomena which evolve through time”.^6^

## Ethics, consent and legal issues

Detailed case studies such as the one presented here are unusual—in part because publishing an article about such involvement can raise complex ethical and legal issues. However, the need for transparency is also well recognised by the Court of Protection and as the judge explicitly acknowledged in his published final judgment in this case: “Decisions of this magnitude, even where they reflect medical and family agreement, require that they be available for public scrutiny, they concern us all” (para. 22).

Everyone who informed this article has been provided with the opportunity to give feedback on it and consent has been obtained for all direct quotations. Because we draw on detailed notes made during our attendance at the court hearings, we shared a draft with the two judges involved in the case, neither of whom has objected to its publication. The family members involved (S's mother, brother and daughter) fully support the publication of this article and the use of their words. Miss S's brother, for example, wrote: “I am more than happy for my quotes to be used”, adding, “I really appreciate the fact that you have used [a particular quote]” [email to authors] and Miss S's mother stated: “I seriously hope that the whole case is eventually in the public domain as [we] have nothing to hide or be ashamed of [and, without public discussion] nothing will change and other families will probably experience some of the devastating aspects we had to endure” [email to authors]. The patient herself, ‘Miss S’, lacked capacity to give consent—but members of her family believe she would have wanted it made public. The court ban on identifying her expired 8 weeks after her death (para. 23), just before publication of this article. We have nonetheless avoided identifying her here as we feel that to do so would not add anything of value to this article.

## Case summary

Our involvement in S's case began in July 2015 when her mother emailed us because she was distraught by the apparent lack of progress in the application to the court for a declaration that it would be lawful to withdraw ANH from her daughter. She wrote: “I hope you don't mind my contacting you personally but the situation we are in is becoming so soul-destroying”. Her daughter, she said, had a PVS diagnosis and the CCG had agreed to take the case to the Court of Protection.^ii^

However, no date had been set for a court hearing even though it was by then three years after her daughter's injury. She explained: “I have been at constant loggerheads…regarding what I view as a failure of duty in the care provided” and “I have lost faith in the ability of [those responsible] to carry out efficiently the process of the application [to court]”. At this point we had a long conversation with S's mother and, at her request, contacted the relevant health and legal teams on her behalf, offering assistance from us and from specialist practitioner colleagues.

The facts are as follows. In August 2012 Miss S suffered a profound hypoglycaemic brain injury after overdosing on gliclazide (whether intentionally or by accident is unknown). She was admitted to hospital unconscious and the Court judgment records that she never again appeared to be aware of herself or her environment (para. 6). Since there was no legally binding Advance Decision to refuse treatment (and no Lasting Power of Attorney for Health and Welfare), decision-making about S's medical treatment lay in the hands of the clinical team. Decisions should have been based on their assessment of S's best interests—an important part of which is what the patient would have wanted in these circumstances in line with her values, wishes, feelings and beliefs (Mental Capacity Act 2005 s4(6)).

By April 2013, S's mother was clear that her daughter would not have wanted any further life-extending treatments and wrote to the clinical team documenting her belief that S would be “absolutely devastated” by her condition: “her quality of life is nil” and “It is my own and her families (*sic*) wishes that her life not be prolonged unnecessarily. […] She has the right to pass away with peace and dignity” (letter given to treating team on 13 April 2013). This should have triggered serious consideration of whether or not it was in S's best interests to continue to receive artificial nutrition and hydration, and, if not, then how to get an application ready for the Court of Protection in a timely and efficient manner. Instead, S (and her family) became trapped in a maze of misinformation, mistakes and procrastination, leading to years of avoidable delay before treatment was finally withdrawn in May 2016 nearly four years after S's injury. This long time lag occurred despite the best efforts of S's family to protect her from life-extending interventions that they believed she would not want, and despite the fact that S could already have been diagnosed as being in a PVS in early 2013.

## Five avoidable delays

**Figure 1:**
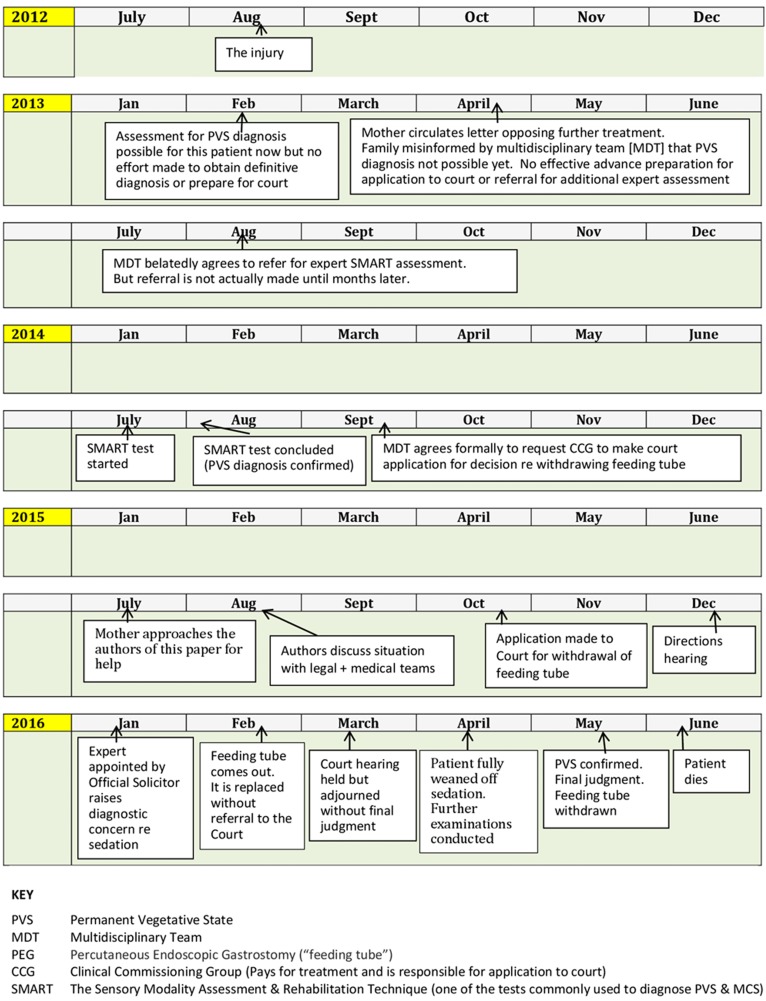
Timeline: summarising key points in background to Cumbria NHS Clinical Commissioning Group (CCG) v Miss S and Ors [2016] EWCOP 32.

**Figure 2:**
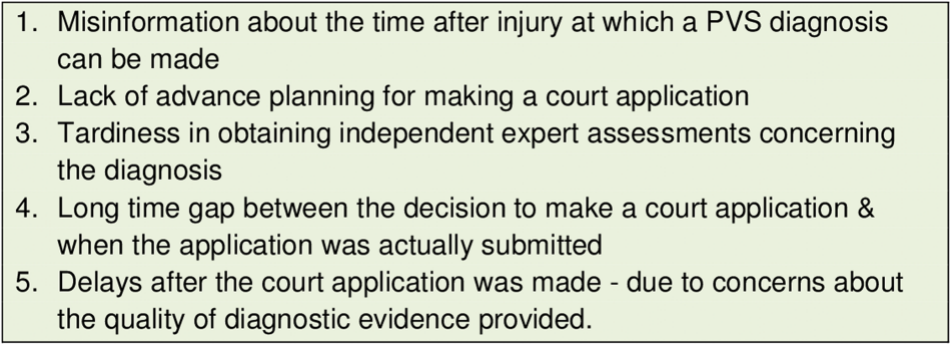
Five key avoidable delay points in the ‘Miss S’ case.

We have identified five key ‘avoidable delays’ contributing to the situation in which, for several years, S was given medical treatment without any evidence that it was in her best interests or anything other than futile. These findings are summarised in figure 1 which presents an overview of the timeline of events and in figure 2 which lists the key delay points in this timeline.

### Avoidable delay 1 was caused by misinformation from professionals about the time after injury at which a PVS diagnosis can be made

In the face of family requests to let S “pass away with peace and dignity” eight months after her injury, there was a formal best interests meeting (13 April 2013), involving S's family and key members of the health care team, including the Mental Capacity Lead for Cumbria Partnership Trust, a Community Macmillan nurse and the patient's GP. At this meeting the family were told that S could not be diagnosed as being in a PVS until *1 year* after the precipitating event. This is incorrect. The vegetative state cannot be diagnosed as permanent until 1 year after a ‘*traumatic*’ injury (eg, a blow to the head)—but this was not the right timescale for S, who had a ‘non-traumatic’ (anoxic or other metabolic) injury, meaning that she could potentially have been diagnosed as permanently vegetative after 6 months.^iii^ At this stage, the clinical team could have been compiling the necessary evidence for a court application. Instead, the ‘action plan’ in the minutes from the best interests meeting was to review diagnosis ‘closer to the 12 month stage’.

### Avoidable delay 2 resulted from lack of advance planning for the legal application

Not only was the clinical team wrong about the timescale for diagnosing the vegetative state as ‘permanent’ for this patient, they also failed to plan ahead for the application to the Court of Protection. Even believing (incorrectly) that S could not be diagnosed as PVS until a year post-injury, they could nonetheless—given the views expressed by S's mother—have begun initial preparations for a court application and booked the relevant tests for the time at which they believed a firm diagnosis might be possible. This was not done. Instead a ‘wait and see’ and ‘plan another meeting/review’ approach appears to have been adopted with no sense of urgency in ensuring that the treatment being provided was in her best interests.

### Avoidable delay 3 was inexplicable tardiness in obtaining independent expert assessments to confirm the patient's diagnosis

Although some tests (including the Wessex Head Injury Matrix) had been done between 6 and 12 months post-injury (and had indicated nothing higher than a reflexive response level), the additional expert diagnostic assessments the Court would need were not carried out until July–August 2014—*2 years* post-injury. This was in spite of there being no clinical reason for the delay and in spite of the family pressing for such assessments to take place as soon as possible. It was only after the results of this assessment (using the Sensory Modality Assessment and Rehabilitation Technique (SMART)) that S's multi-disciplinary team (MDT) agreed to ask the CCG to make an application to court. Tardiness in obtaining these tests meant that the team did not make this request until September 2014, more than two years after the initial injury.

### Avoidable delay 4 was a delay of more than a year between the decision to make a court application and the date when the application was actually made

Although the multi-disciplinary team agreed on 18 September 2014 formally to request that the CCG make an application to the Court of Protection, this application was not actually made until October 2015, 13 months later. We are not sure when the CCG formally received that request, or when the solicitors were instructed (so it is possible that some of the delay may have been introduced by either the treating team or the funding commissioning organisation) but we do know that the case had been in the hands of the CCG solicitors for some time when S's family approached us for help in mid-July 2015—at which point we contacted the CCG solicitors directly to try to find out (on behalf of the family) what progress had been made. In subsequent correspondence, one of the solicitors acting for the CCG explained why the process of preparing an application can be so time-consuming:

A difficulty that can be encountered with regard to acting for CCGs in particular is that, whilst they fund and commission the care, the care providers are separate organisations and the CCG therefore has no direct control over the medical records, and further none of the treating clinicians or medical practitioners are employees of the CCG. This means that the CCG has a coordinating role with numerous other organisations behind the scenes and it is necessary to liaise with each of these numerous organisations, such as multiple NHS Trusts [the letter names three for this case], GP surgeries, hospitals/nursing homes etc, to obtain the relevant records and to undertake investigations, which can be a time-consuming and unwieldy process. (Letter to authors from CCG's solicitor, 18 July 2016)

The CCG's solicitor also told us that work on preparing the case for court had been halted for some months while S was ill. The solicitors say this was occasioned by the nurses caring for S who had been asked to prepare statements for the application: they “contacted the CCG and explained that they would have to delay preparing their statements as S was very unwell and their time was needed to provide care, notwithstanding that the outcome for S was unclear” (letter to authors from CCG's solicitor, 18 July 2016). (Note: we do not have access to the nurses' perspective on this issue and it is not clear to us why they were not provided with the support necessary to complete this task in a timely manner.)

Given that this was a relatively straightforward case (with no disagreement between family and clinicians) one might reasonably expect completion of the paperwork for the court application to take no more than a few months (as it has done in other cases eg, *Re D* discussed below). The 13-month gap between the MDT's decision to refer to Court and the lodging of the application is simply unconscionable.

### Avoidable delay 5: adjournment of the March 2016 hearing: an additional 2-month delay resulting from concerns about the quality of diagnostic evidence provided to the court

Once the application had been made in October 2015, the judges involved approached proceedings with a clear sense of urgency. This was evident even at the directions hearing (December 2015) when the judge, Jackson J, listened to the family's concerns about the treatment of S and the protracted journey that had finally bought the case to court and made clear statements about the need to proceed efficiently from this point. In an effort to avoid further delays he sought assurances that the Official Solicitor would instruct his own independent expert only if review of the medical records suggested there was good reason to do so. He also directed that any such report must be filed by early February to allow a swift move to a final hearing as soon as possible thereafter. The directions hearing was an important milestone for the family. S's mother said as she left the hearing: “So, at least there is a time scale now—no decision, but at least a time scale. The judge listened. I feel it's been a good day”.

In the event, however, the legal proceedings were to take 8 months from application to final judgment—involving another (fifth) layer of delay in deciding the outcome for S, with particularly distressing consequences for S's family. Hopes of a speedy process through the Court began to unravel once the Official Solicitor reviewed the evidence provided by the CCG and decided to instruct his own expert—particularly to look at whether the drugs being given to the patient might have had a sedative effect which interfered with an accurate diagnosis (para 2(8)). Negotiations in January 2016 between this expert and S's consultant led to an agreement that it was appropriate to wean her off sedation to allow further testing of her diagnosis—although this had not, in the consultant's earlier view, been necessary or in her best interests. The family then faced an anxious wait prior to the hearing—scheduled for March 2016—with the threat of further adjournment, which was now requested by the CCG solicitors on the grounds that the CCG no longer felt it had adequate evidence to support its original case (made on the basis of a diagnosis of PVS).

This time period was particularly stressful for the family because—in February 2016—S's feeding tube (a Percutaneous Endoscopic Gastrostomy (PEG)) became nonfunctional. It was replaced in the face of family opposition and without taking the decision back to the Court of Protection.^iv^ Replacing the tube felt ‘like assault’ to members of S's family—and was particularly distressing for her brother and her teenage daughter, both of whom felt that S's body had ‘rejected’ the feeding tube and that this was an opportunity to ‘let nature to takes its course’ without implicating anyone in S's death by actually *withdrawing* the tube (a painful decision to be involved in for family members even when they believe it is the correct one).^4^ As S's brother explained:

That PEG tube perishing, it's kind of like it's natural, nobody's forced it, nobody's had to make a decision, it's just nature taking its course. This is the perfect opportunity just to, you know, start the process, let her go. But no, we had to reinsert it.

The family became increasingly desperate after this. S's teenage daughter (who supported the court application) had originally hoped not to be actively implicated in it. However, she was the only family member eligible for legal aid and she now felt pushed into a position where she had to become directly involved, first in the application for legal aid to fund ongoing legal representation and then in attending the hearing. She also wrote a letter to the judge about her mother: “It is wrong and cruel to keep her body alive”, she wrote, “please respect her wishes and let her be at peace”.

From the family's point of view this was an ‘avoidable’ delay. S's mother, brother and daughter were adamant that S would have wished to refuse life-extending treatment irrespective of the diagnostic label attached to her condition. As S's mother stated in a letter to the judge:

I do not think that it would make any difference to [my daughter] if she was diagnosed as being in a persistent vegetative state or a minimally conscious state. Either way, she would consider that she does not have any quality of life. […] I have no doubt that she would not wish to continue living in this condition.

At the March hearing, the judge, Hayden J, clearly took a great deal of notice of this point of view (a fact much appreciated by the family). He reiterated their arguments from the bench, including acknowledging that S's best interests might not be the same as fine-tuning the diagnosis. He also expressed concern about delays—stating (according to our notes taken during attendance at this hearing) that: “inordinate, unjustified delay is inimical to S's best interests *whatever* the outcome”. Nevertheless, in the end he ‘reluctantly’ concluded that the court was left “with very little alternative…given the state of the expert evidence” (para. 14) but to adjourn until May to allow sedation to be reduced and further examinations to be carried out.

The judge made every effort to ensure a tight timetable at this point. He ordered both of the experts who had already provided a PVS diagnosis to have a telephone conference with the Official Solicitor's chosen expert the next day and to put in place a clear care plan to allow further assessments to be carried out within 2 months.

The 2-month adjournment was presented by the judge as ‘not long’ (especially in light of how long proceedings had taken already). However, it was devastating for the three generations of S's family who had already endured an extremely difficult few months since the directions hearing and had travelled up to Liverpool for the March hearing, hoping that a final decision might be made, as originally anticipated. Her brother came out of the hearing shocked by the way the legal discussion had developed. He felt the court had ‘lost sight’ of his sister as a person:

The court case [at first] was all about S's best interests until [the consultant appointed by the Official Solicitor] came into it. And then the whole proceedings were taken over about was she minimally aware or was she PVS. When just before that we were talking about her best interests. So S flipped from being a human being to a case study in my opinion.

He was particularly disturbed that an expert who had not even visited his sister, or seen all her notes, was, in his view, able to have such an effect on proceedings:

The influence that [the Official Solicitor's expert] had on the Court shocked me, because it changed the whole dynamic of the conversations that were going on. And I was looking at my mam [mother] thinking: “we're not talking about [my sister] anymore, we're talking about somebody who they want to get a definitive diagnosis from”.

The adjournment meant that, instead of a resolution, the family faced ongoing limbo, with no guarantee that a final judgment would be made even at the May hearing if, at that point, the diagnosis were still in question. For S's mother, in particular, after years of being told that all tests showed her daughter had no awareness, the suggestion that this might not after all be true was profoundly distressing. The subsequent weeks of waiting for further confirmation of her daughter's diagnosis were ‘horrendous’—in part because she was horrified to think that S might be feeling pain and distress (especially with sedation reduced). She felt her daughter was being treated as ‘an experiment, not a person’ adding: “They've forgotten about the patient. I'm praying for a miracle—may *compassion* triumph”. In the meantime, sitting at her daughter's bedside during these weeks between March and May 2016 she always left the door open so staff could see her, because “you do think about the pillow over the face…you do”.^v^

The short, but highly distressing adjournment to address questions about the security of the PVS diagnosis was a ‘clinically purposive’ delay—in that its purpose was to seek further diagnostic precision and certainty—but it was nonetheless ‘avoidable’ in that the sedation issue could perhaps have been resolved *prior* to the case reaching court and in parallel to other processes (eg, if the sedation issue had been discussed and a way forward agreed with the Official Solicitor prior to the directions hearing). Given that the SMART test confirming PVS was carried out in August 2014, there was obviously time, and there surely *could* have been a mechanism, for questions to be raised and addressed prior to the directions hearing in December 2015. A more fundamental issue, however, concerns how cases for ANH-withdrawal from patients in disorders of consciousness are framed and judged and the extent to which they hang on determining precisely where a patient's diagnosis lies on an often-contested or ‘illusory’ continuum.^7^ ^8^ Bringing the case for withdrawal on the basis that the patient has a PVS diagnosis may have contributed to avoidable delays, even though the PVS diagnosis was ultimately judged to be secure. This opens up a whole new discussion about the extent to which a definitive diagnostic label is necessary to determine best interests—an issue too extensive to review here, but which we will address in another paper.

As it turned out, the newly commissioned tests, like all the tests before them, indicated that S was in a PVS. Finally, 3 years and nine months after her initial brain injury, the judge ruled that ANH was not in S's best interests and could be withdrawn. She died on 4th June 2016. According to her mother, “it was very peaceful”.

Having identified five causes of avoidable delay for this patient, we now compare this case with others concerning ANH withdrawal from patients in PVS, address how delays might be avoided in future and reflect on the way forward.

## Some comparisons with other cases

The length of time this case took to be resolved is sadly rather typical of many cases that go through the courts. We have noted in a previous study that clinical teams may not take the initiative to ensure timely best interests decision-making and we have highlighted the extent to which families who come to believe their relative would want treatment withdrawn find themselves having to lobby on behalf of the patient.^4^ ^5^ Many of the factors that delayed the court application in the case of Miss S are common to other cases: misinformation, lack of advance planning, delays in amassing medical records and statements from across different organisations and care providers. The Miss S case also captures two not-uncommon incidents which create crises for families over and above the day-to-day, year-to-year trauma of witnessing a relative subject to treatment it is believed the patient would not want: (i) the PEG failing, and being replaced, while proceedings to obtain a court judgment to remove it are in process and (ii) belated disputes about diagnosis, which are particularly distressing if family members believe—as in this case—that being minimally conscious would make the situation still more unbearable.^vi^

However, the trajectories of cases coming to the courts need not be subject to these delays, nor result in such distressing crisis incidents. We can compare S's case with a couple of (unusually efficient) recent instances where applications were made in a smooth and timely manner. Both concerned patients (like S) with non-traumatic brain injuries and in both cases final judgments to withdraw ANH were achieved *less than a year* after injury.

- *Re D* [2012] EWHC 885 (COP) the injury occurred on 25 July 2011 and—after extensive testing—a best interests meeting took place just *two months* later (September 2011) at which it was agreed to make an application to the Court of Protection for treatment withdrawal. The application—supported by detailed neurological evidence from experts—was made in December 2011 (5 months post-injury). The case was heard (with up-to-date tests done at the 6 months point) and withdrawal was approved at the end of March 2012, that is, a period of 8 months from injury to treatment withdrawal. (One distinctive feature of this case which may have contributed to the relatively speedy resolution was that the patient had written a purported—though invalid—advance decision to refuse treatment).
- In another recent case (unreported) the Trust sought advice from solicitors within 6 weeks of the patient's injury and followed the advice they were given to conduct testing within the 6-month period and make an early application to the Court (5 months post-injury) supported by expert neurological evidence of the patient's diagnosis and prognosis, and with up-to-date review at the 6 month point: this again resulted in a period of 8 months from injury to court approval of the lawfulness of treatment-withdrawal.

Although every case has distinctive characteristics and may involve its own specific challenges (eg, whether or not the patient's care is being paid for and delivered within the same provider system), there appear to be no significant differences between these cases and the case of Miss S in relation to two key features: in all three the eventual diagnosis (PVS) was the same, and in all three the family and the treating clinicians shared the same view as to the patient's best interests. There is, then, no ‘in principle’ reason why Miss S's case could not have followed a trajectory and timescale similar to these other two. According to a specialist in neurological rehabilitation who acted as expert witness in court there is no reason why she could not have been diagnosed as PVS in February 2013 (since interventions which can get in the way of assessing consciousness—eg, ventilation and tracheostomy—had been removed by then) and it would certainly have been possible to have a PVS diagnosis and start preparing an application in April that year—the point at which her mother wrote the letter pleading that “her life not be prolonged unnecessarily”.

**Figure 3:**
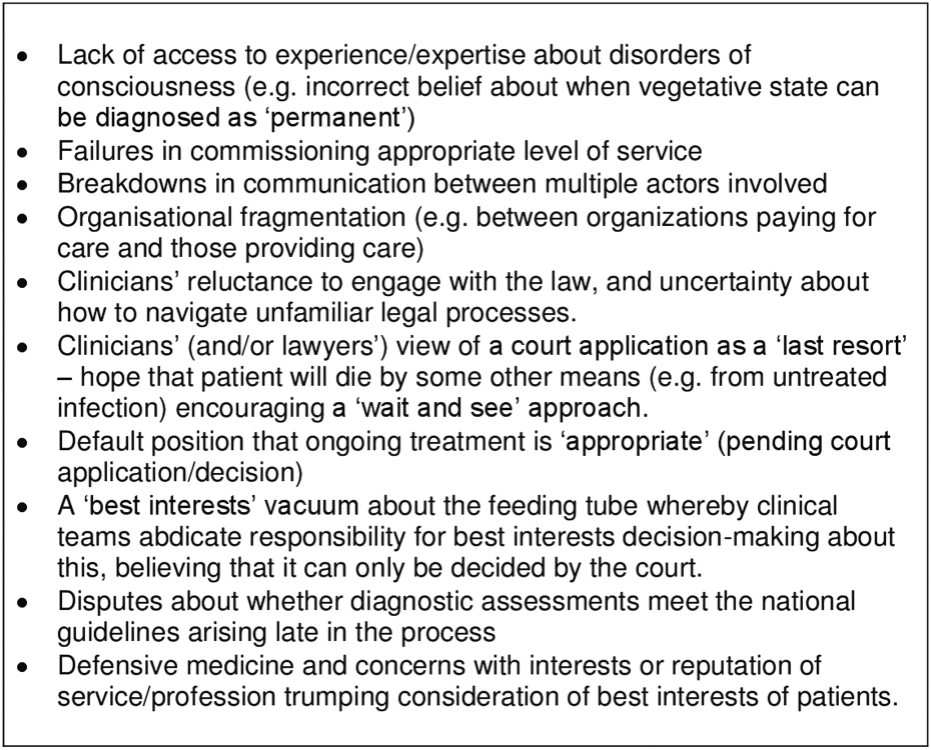
Factors that may obstruct timely withdrawal of the feeding tube from a permanent vegetative state patient when family and treating team agree that treatment is not in the patient's best interests.

Insofar as delays for Miss S were caused by practical considerations not relevant in the other two cases (such as locating full medical records or obtaining statements), CCGs need to take responsibility for identifying these causes of delay and for finding practical solutions. In our earlier work^4^ ^5^ we reported that some families fighting to bring applications to court believe that proceedings have been deliberately protracted or obstructed because of clinicians' religious or ethical objections to treatment-withdrawal, but we have no evidence to suggest this was so in this case. Instead, delays seemed to have been caused by a combination of: system fragmentation in the delivery of care; lack of access to appropriate medical and legal expertise at key points; a disjointed and ineffective system administered by professionals who did not adequately understand—and/or were inadequately supported within—the system; a lack of resilience in the system to deal with difficulties as they occurred; communication breakdowns; and problems with the level of service commissioned, especially given that prolonged disorders of consciousness are relatively unusual (and cases for ANH withdrawal even more so) such that most medical and legal teams lack experience in this area (see figure 3).

In a lengthy conversation with S's consultant after the case was concluded, he identified problems in his own handling of the situation and with the way in which the CCG handled making the application. He also told us that he felt that the need to go to court had got in the way of, rather than promoted, his patient's best interests. He felt guilty about what had happened to S but said that he was under a lot of pressure and sometimes gave priority to “more pressing concerns” and “did not chase things as I should have”. He believed that, due to the level of service commissioned, “we were not working as a multi-disciplinary team who could meet regularly as we should do”. He added, “it comes down to the commissioning. If you don't commission a service you rely on good will”. He also felt his own knowledge of the patient, based on having looked after her for several years, was given less status than that of the expert witnesses called on by the Court, and that his expertise was marginalised. In retrospect he wishes he had stood his ground in asserting what he believed to be in his patient's best interests—and that he had attended the March 2016 hearing in person and defended this position. It is impossible to know what effect this would have had, but is worth noting that, in the course of that hearing, the judge commented that S's consultant appeared to have ‘yielded’ to the view of the Official Solicitor's expert about the appropriate course of action rather than continuing to assert his own view of his patient's best interests, and had perhaps ‘fallen on his sword’ unnecessarily (taken from our notes from the hearing).

## Avoiding delays: potential solutions and the way forward

The costs of continuing to give treatment that is not in the patient's best interests (and which the family believes the patient would not want) are huge, both financially and ethically. Financially, the cost of keeping each PVS patient alive is around £90 000 per year: this displaces alternative NHS services and causes a loss of seven quality-adjusted life years from other NHS patients who are in a position to benefit from treatment.^9^ We estimate that the avoidable delays we have described in relation to this particular case resulted in an additional 3 years of unwanted/futile treatment—at a cost of £270 000 or 21 quality-adjusted life years for other NHS patients. (This was on top of the legal bills for the NHS, the costs of incidents such as hospitalisation for PEG replacement, and the cost of legal aid for the family).

Ethically, the delays resulted in S, the person ‘at the centre’ of this story (para. 13), being subjected for years to treatment that she would not have wanted and which was not in her best interests. In addition her family was subject to years of distress, and to an unnecessarily agonising series of delays during the course of the court hearings. The delays meant they had to do much more than simply bear witness to S's wishes, values and beliefs (as required by the Mental Capacity Act 2005); instead they were forced into the position of becoming active advocates for withdrawing the feeding tube—potentially leaving them feeling burdened, entirely wrongly, with responsibility for ‘causing’ her death. The whole situation also caused unnecessary stress to staff (including contributing to tensions between the family and S's health care team) and triggered moral distress too. Once they come to believe that life-extending treatment is not in the patient's best interests, staff charged with continuing to deliver treatment can experience their work as ethically problematic—still more so if they are actively involved in reinserting or replacing feeding tubes under these circumstances. Legally, giving treatment that is not in the patient's best interests may be regarded as assault or battery (*Bland* [1993] AC 789 per Lord Browne-Wilkinson at para. 883). In an important ruling subsequent to the Mental Capacity Act, it was clearly stated that it is not lawful to give treatment that is not in the patient's best interests:

[T]he focus is on whether it is in the patient's best interests to give the treatment, rather than on whether it is in his best interests to withhold or withdraw it. If the treatment is not in his best interests, the court will not be able to give its consent on his behalf and it will follow that it will be lawful to withhold or withdraw it. *Indeed, it will follow that it will not be lawful to give it*. (*Aintree* [2013] UKSC 67)

It is not surprising that some staff, as well as family members, were deeply troubled by what was done to Miss S.

Our case study approach in this paper suggests that efforts to solve the problem of delays could focus on three areas. First, it is important to improve the way those who fund and provide care consider best interests in relation to ANH-provision and—also, where necessary/appropriate—how they then refer cases and prepare applications for withdrawal. Second, if we work with the widespread assumption that court applications are required before ANH can be lawfully withdrawn from patients in permanent vegetative or minimally conscious states, then there needs to be careful consideration given to legal practice in relation to ‘best interests’ and the role of the Official Solicitor in considering the weight to be placed on patients' own wishes. Third, at a more fundamental level, there is the need to question the perceived requirement for a court application to ‘authorise’ withdrawal from such patients—since this lies at the root of ‘avoidable delays’. We will consider each in turn.

### Provision and inspection of care for patients in prolonged disorders of consciousness

It would be helpful to create a register of patients in prolonged disorders of consciousness in order to ensure that appropriate specialist resources and follow-up are available for them and to ensure that those who fund and provide care for these patients (and their legal teams) are aware of the national clinical guidelines, including timetables for action. It would also be important to provide access to expert co-ordination (eg, perhaps a case manager focused on timely best interests decision-making)^10^ and give support and training. In addition, official monitoring processes might also usefully incorporate systems that foreground best interests. Annual reviews of continuing health care funding, and regular inspections of care homes, do not currently routinely check that the ANH being provided to the patient is in their best interests. If either of these routine inspections were to flag up such issues, court applications might be started much sooner. Despite the fact that no court in England and Wales has ever found ANH to be in the best interests of PVS patients, such treatment is routine and is delivered to up to 16 000 PVS patients in the UK^11^ on a daily basis. This practice is supported by an organisational infrastructure which includes CCGs, insurance companies, and NHS continuing healthcare funding as well as the (largely ‘independent sector’) business of long-term care home provision. Those delivering, paying for, or receiving payment for, the treatment of PVS patients with ANH do not seem to fear prosecution for offences against the person, and the law seems tacitly to condone this. It may be that attention could be focused—and action galvanised—if a legal case for battery were to be brought against those who commission or provide on-going treatment to PVS patients without ensuring timely expert assessments and decision-making about whether that treatment is actually in the patient's best interests.^12^ A case could also be made for breach of Article 8 of the European Convention on Human Rights on the basis that treatment represents an unlawful interference with the patient's autonomy.

### Legal practice in relation to ‘best interests’

Legal practice in relation to ‘best interests’ and the weight to be placed on the patients' own wishes is the second key area where efforts to solve the problems highlighted in this case study might focus. There is of course no definition in the Mental Capacity Act as to the priority (or otherwise) to be given to the person's own wishes in determining their ‘best interests’—it is one factor among others. Several post-*Aintree* cases show that the judiciary of the Court of Protection are increasingly willing to engage with the wishes of the individual, even when weighed against ‘sanctity of life’ considerations, but “at law the weight placed on the person's wishes, feelings, values and beliefs still remains largely within the discretion of the best interests decision maker: and the Mental Capacity Act 2005 does not require an explicit justification for acting contrary to them”.^13^

The role of the Official Solicitor in applications to the Court of Protection has become a cause for concern with the suggestion that he is more “foe” than “litigation friend” to those who may lack capacity in so far as he advocates not for what the patient wants (or would have wanted) but for what he independently considers to be in the patient's ‘best interests’ (in accordance with s5 of the Mental Capacity Act 2005). This can sometimes, as in this case, appear (eg, to the patient's family) as if the patient's wishes, feelings, values and beliefs are being sidelined and it can create an ‘adversarial’ context for decision-making. In particular, the perceived likelihood that the Official Solicitor will oppose treatment-withdrawal in all but the most clear-cut cases (regardless of reports of the patient's own wishes) creates a context in which continued treatment becomes the default option.

In addition, it has been widely noted that the ‘best interests’ standard is not compliant with the UN Committee on the Rights of Persons with Disabilities, and discussions conducted as part of the Essex Autonomy Project have proposed as “a good point of departure” that there should be a “defeasible presumption that actions taken in the best interests of P requires making decisions that achieve the outcome that P would prefer”.^15^ The Law Commission has recently proposed that section 4 of the Mental Capacity Act 2005 “should be amended to establish that decision-makers should begin with the assumption that the person's past and present wishes and feelings should be determinative of the best interests decision”. This development would offer an important opportunity to shift the current focus of court cases about patients in prolonged disorders of consciousness from a preoccupation with the person's diagnosis and prognosis, to a full consideration of what the person would have wanted (as testified by those who knew them).

### The perceived requirement for a court application

The perceived requirement for a court application lies at the root of ‘avoidable delays’. Although the CCG that commissions care bears ultimate responsibility for many of the delays we identified in relation to S, these delays were shaped by the perceived need to go to court. Instead of the clinical team consulting with those close to the patient and making and enacting the best interests decision as they do with other patients, they became enmeshed with lawyers and legal procedures. This case study highlights how going to court can distance the team caring for the patient from decision-making and trigger complex lines of communication and decision–making (eg, divisions between who is providing care and who is commissioning care). The (apparent) need to go to court to withdraw such treatment conveys a message that providing ANH is the correct position unless and until a court rules otherwise. This can lead to an abdication of best interests decision-making by the clinical team, who may feel that it has been placed outside their jurisdiction. Going to court necessarily causes a time lag between clinicians coming to the conclusion that treatment is not in the patient's best interests, and actually being able to act in accordance with this assessment. Clinicians may therefore be left feeling that decision-making about ANH is not an urgent issue to resolve; alternatively, they seek to circumvent the need to go to court by trying to allow the patient to die via decisions they believe they *are* able lawfully to make without court applications, such as withholding antibiotics. A court application will always, of necessity, involve a further time lag in the process (between application and judgment)—but this case study illustrates how the delay it creates can stretch backward into the process, creating avoidable delays long before the application is ever made (see figure 3).

**Figure 4:**
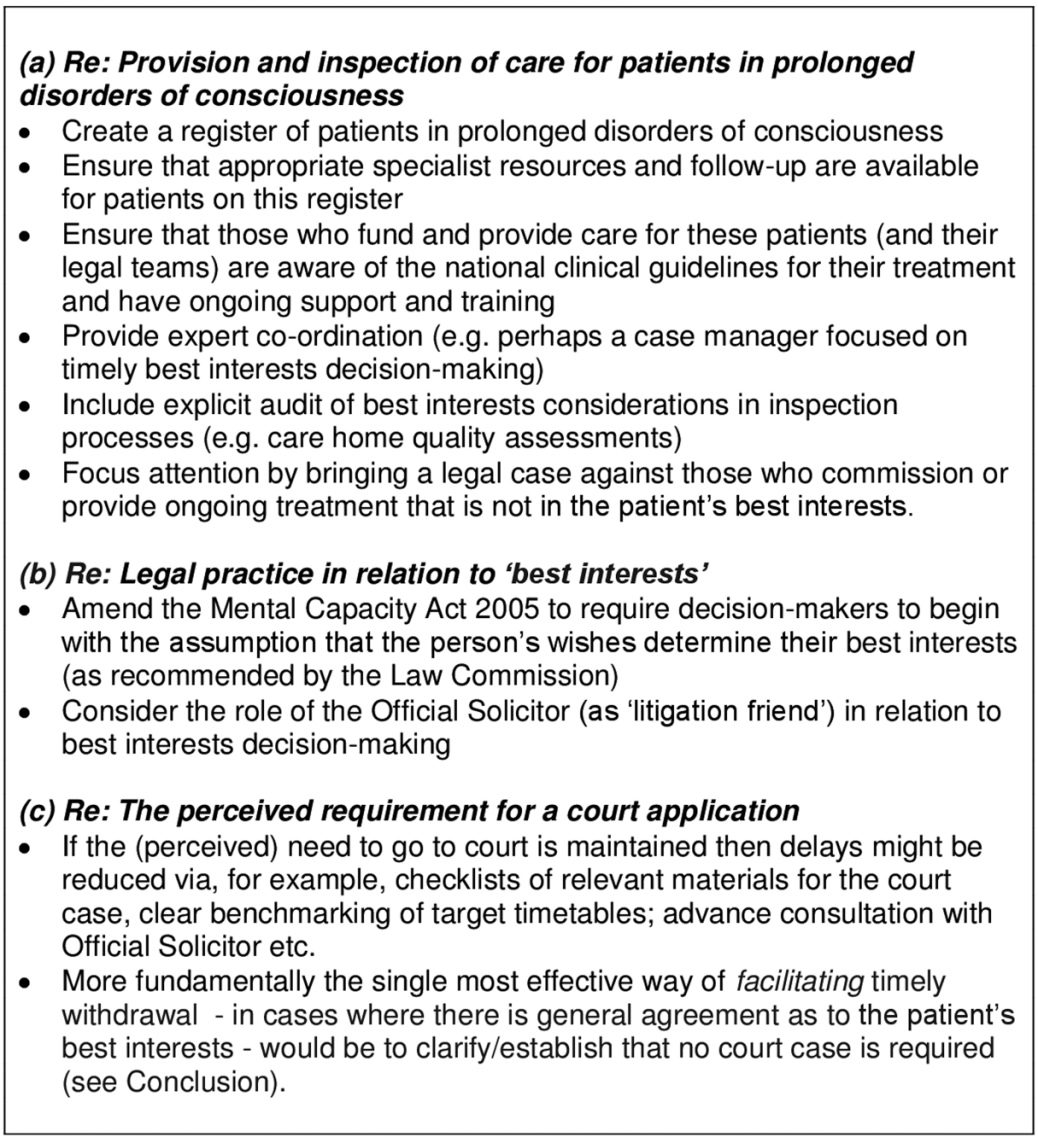
Factors that may facilitate timely withdrawal of the feeding tube from a permanent vegetative state patient when family and treating team agree that treatment is not in the patient's best interests.

In addition, the Court seems recently to have inadvertently caused delays as an unintended consequence of rebukes to solicitors representing CCGs for failing to prepare cases well. Recent case law (*Sheffield Teaching Hospitals NHS Foundation Trust v TH*; *St George's Healthcare NHS Trust v P and Q*) has been critical of applications launched ‘precipitously’ before a secure diagnosis is in place. Despite clear differences between these cases and the consensus withdrawal cases described in this article, legal teams have reacted with a great deal of anxiety about “dotting the ‘i's and ‘crossing the ‘t's’” before bringing a case to court. This contributes to a clear tension insofar as, on the one hand legal teams are keen to ensure that everything the Court might require is put in place before the court hearing, and, on the other hand, their lack of experience (in many cases) with what is in fact required. Current guidance (eg, via Practice Directions to the Court of Protection Rules) is minimal or non-existent. If it continues to be perceived as necessary to take all—or at least some of—these cases to court, then a checklist of relevant materials and access to expert advice for CCG legal teams would be extremely beneficial. This could be alongside clear benchmarking about the target timetable for preparing the application to court, advance consultation with the Official Solicitor and/or a pool of agreed independent experts to confirm diagnoses (with a limit on the number to be involved unless special circumstances pertain). Factors that may facilitate timely treatment-withdrawal are displayed in figure 4.

## Conclusion

The continued provision of life-prolonging treatment for patients in PVS—regardless of their prior expressed wishes—is widely acknowledged as a problem and a range of solutions has been offered, each of which displaces the problem onto different stakeholders. One solution is for individuals who would not want to be kept alive in PVS to write Advance Decisions refusing life-prolonging treatment in the event of ending up in such a condition;^16^ ^17^ another is for better use to be made of the ‘window of opportunity’ for death^4^ at different points along the trajectory that results in such states (eg, death via withholding or withdrawing treatment in critical care). Collectively, the body of work of which this article is a part addresses these different proposed solutions and explores the interface between them. On the basis of this particular case study, however, we suggest that the single most effective way of addressing the problem of ‘avoidable delays’ in withdrawing unwanted/futile treatment from existing PVS patients would be to abolish the (perceived) need for a court hearing in cases where there is general agreement (as in the case examined here) as to the patient's best interests. We are hopeful that this may be addressed in the next iteration of the Practice Directions and that it will be made clear that doctors are not under a legal duty to seek the court's approval before withdrawing ANH. Recent legal analysis questions whether (as the current iteration of Practice Direction 9E to the Court of Protection Rules suggests) it is really legally required for doctors to seek the approval of the court before they can lawfully withdraw ANH from patients in disorders of consciousness: the conclusion is that “It seems to be plain that it is not, and cannot be”.^18^

If it were made clear that—for PVS as for other patients—doctors were able (following assessments properly compliant with the Mental Capacity Act 2005) to withdraw treatment not in the patient's best interests without recourse to the courts, many of the delays identified in this case study could be avoided. We would then need revised Guidelines from the Royal College of Physicians and training for clinicians and education for lawyers to ensure that treatment is withdrawn in a timely manner. Appropriate support (as suggested above) will also need to be available to clinicians and legal teams to expedite those court applications that *are* still required where there is doubt about the patient's best interests. (The cases that *will* still need to go to court would benefit from the extra court time and expertise released via this strategy too). Returning treatment-withdrawal from patients in prolonged disorders of consciousness to normal medico-legal practice, and allowing it to follow the same best interests procedures as are employed for all other patients (without valid and applicable Advance Decisions to refuse treatment) does not mean that such decisions will be trouble-free in future. They will be subject to all the same difficulties that beset best interests decision-making generally, but released from the special problems currently imposed by the perceived requirement for court approval.

Ethical debates concerning provision of ANH to PVS patients have been well rehearsed in the quarter century since *Bland* (eg, in this journal).^5^ ^7^ ^17^ ^19–21^ In the particular case analysed here, it should have been straightforward. No new ethical or legal issues were at stake and every expert who examined Miss S—before and after sedation had been reduced—agreed she was in PVS. Ultimately the court judgment concerning S was uncontroversial and has, at the time of publication, attracted very little attention, either in the mass media or in on-line forums where the ethics of end-of-life decision-making are debated. The family are determined to make their story public and we are hoping this article will help inform further public and professional debate once it is published (We are also grateful to the Transparency Project at Cardiff University for putting us in touch with a journalist who might be interested in covering the story).

This mundane account of a catalogue of delays and of the complex dynamics between medical and legal practices is unlikely to capture the imagination of bioethicists. There is no central moral conundrum, no grand theoretical divergence between opposing parties. Yet this is the ordinary quotidian reality for PVS patients, their families, and their health care teams. The ‘devil is in the detail’ that constitutes the context in which practical ethical decision-making concerning these patients must be addressed and implemented—in relation to which we must identify solutions to avoid breaching the human rights of these patients in future. There is a clear need for intensive bioethical engagement with these issues and for those who provide care for, and frame the law about, these patients, to respond to the challenges identified.

## Footnotes

i We had recently chaired an expert Working Party on Practice Direction 9E to the Court of Protection Rules 2007, on the basis of which we wrote to the Court of Protection Rules Committee recommending that treatment withdrawal from patients in prolonged disorders of consciousness should follow the legal and professional guidelines laid down for all other patients, permitting withdrawal without recourse to the courts if clinicians determine that it is not in the patient's best interests (and if this is not contested). We also expressed concern about the length of time it was taking for cases such as these to be resolved by the courts and suggested ways of streamlining the process. We await their response.

ii Since *Bland* (*Airedale NHS Trust v Bland* [1993] 1 All ER 821 HL) an application to the Court of Protection has been assumed to be a legal requirement before ANH can be withdrawn from patients in PVS. It is presented as a matter of good practice by the Mental Capacity Act Code of Practice in England and Wales and this is also stated in Practice Direction 9E to the Court of Protection Rules 2007 and the national clinical guidelines—but all three of these documents involve a contested interpretation of the law.

iii Royal College of Physicians (RCP) National Guidelines 2013: pp. 10–13. Guidelines in the USA are different—allowing PVS to be diagnosed as early as 3 months after a ‘non-traumatic’ injury.

iv We understand from the CCG solicitors that they were not involved in the provision of legal advice in relation to PEG replacement. This matter was dealt with by solicitors for a different NHS Trust who were aware that there was a pending application with the Court of Protection. The CCG solicitors were ‘surprised’ that the advice provided to the treating clinicians appeared to have been to replace the PEG, despite the opposition of the family.

v Other families we have interviewed describe similar feelings in similar situations, including one man who reports that the whole process of getting to court for his severely brain injured mother had been ‘hideously slow’ but it was the late introduction of a report from the Official Solicitor's appointed expert questioning a PVS diagnosis which ‘was perhaps the most appalling individual event of the whole shebang’. It was this, he believes, that triggered his father's decision to kill the patient and himself.

vi Our broader research shows that for some families the suggestion that their relative might be minimally conscious introduces hope for current, or future, quality of life compatible with what their relative would have wanted, or might come to accept, whereas for others it makes them even more convinced that their relative would want to refuse all life-extending treatment.^2^ This is one reason why best interests of individual patients cannot be determined on the basis of diagnosis alone: for some patients and their families specific diagnosis is highly relevant and for others it is not.
